# Investigation on Damage and Energy Absorption Performance of Aluminum Foam Sandwich Plates Under Low-Velocity Impact

**DOI:** 10.3390/ma19010046

**Published:** 2025-12-22

**Authors:** Kailing Guo, Yunfang Zhu, Shuo Zhou, Ling Zhu

**Affiliations:** 1 Key Laboratory of High Performance Ship Technology, Wuhan University of Technology, Ministry of Education, Wuhan 430063, China; guokailing@whut.edu.cn; 2 School of Naval Architecture, Ocean and Energy Power Engineering, Wuhan University of Technology, Wuhan 430063, China; zyf0930@whut.edu.cn (Y.Z.); 298288@whut.edu.cn (S.Z.)

**Keywords:** aluminum foam sandwich plates, penetration response, impact energy, impactor diameter, ambient temperature

## Abstract

Marine structures may suffer collision during navigation, leading to plastic deformation or even fracture failure of the structure, which poses a serious threat to ship structural safety. In this study, INSTRON 9350 Drop Tower was employed to carry out the impact test on the aluminum foam sandwich plates (AFSPs). The penetration performance of AFSPs were analyzed, including deformation mode, failure mode, impact force, displacement, energy absorption, and loading–unloading process. Additionally, the effects of impactor diameter and low-temperature environment on the penetration behavior of AFSPs were explored. The results indicate that the upper face sheet primarily exhibits shear failure, while the lower face sheet mainly undergoes global bending and tensile fracture. As the impact energy increases, the deformation zone of the lower face sheet extends to the boundary of the effective area of the sandwich plates. The loading stage of AFSPs under different impact energies generally coincide, but the unloading stage shows significant differences. Moreover, the peak impact force of the case D40 is nearly twice that of the case D25, while the 25 mm impactor is more likely to penetrate the lower face sheet, so that the energy absorption of the smaller impactor is reduced. Under penetration conditions, higher impact energies resulted in faster energy absorption rates, but the final absorbed energy values were almost identical. Ambient temperature affects the penetration performance of AFSPs; as the temperature decreases, the permanent deflection of the upper face sheet and the rebound velocity of the impactor decrease, whereas energy absorption increases. Compared with the normal temperature (20 °C), the energy absorption increases by about 8% at low temperature (−60 °C).

## 1. Introduction

Ships may often experience collision from other objects during navigation. For instance, polar vessels are susceptible to floating ice impacts, and ships in busy waterways face mutual collision risks. These impact loads can induce plastic deformation or even structural failure, seriously compromising the ship’s safety.

Extensive research has demonstrated that aluminum foam sandwich structures exhibit high specific strength, high specific stiffness, and excellent energy absorption properties, showing broad application prospects in ship and ocean engineering. Crupi et al. [1] conducted low-velocity impact tests on aluminum foam and honeycomb sandwich plates, comparing their dynamic responses and evaluating their suitability in ship collision protection design. Epasto et al. [2] investigated the effects of core thickness, face sheet materials, and impact energy on different types of AFSPs under low-velocity impact tests. Guo et al. [3,4,5] experimentally studied the dynamic responses of porous sandwich structures, comparing the impact resistance of AFSPs and balsa wood sandwich plates (BWSPs); repeated impact behavior of foam-core beams and plates was also analyzed, and impactor shape effects was examined. Breuning et al. [6] performed dynamic impact tests on foam-core composite specimens, and damage mechanisms under varying impact loads were revealed. Damghani et al. [7] conducted drop weight impact testing machine to study the influence of impactor mass, velocity, and foam density on plate deformation and energy attenuation. Ang et al. [8] employed finite element simulations to investigate the energy absorption performance of sandwich plates, and explored the method to enhance the impact resistance of sandwich plates in ship collisions. DiBella et al. [9] evaluated the energy absorption capacity of sandwich structures through low-velocity impact tests, and the comparison of energy absorption values confirmed their superior energy-absorbing performance, demonstrating that sandwich structures are a promising and sustainable alternative for marine structures. Mozafari et al. [10] quantified the energy absorption performance of closed-cell aluminum foam during impact and proposed a metal foam sleeve (MFS) device suitable for collision protection in offshore wind turbine piles. Epasto et al. [11] optimized lightweight AFSPs through impact tests and introduced a novel protection mechanism (metal foam shell-MFS). Guo et al. [12] systematically employed experimental, theoretical, and numerical methods for analyzing sandwich structures under impact, and demonstrated that the sandwich structure has good impact resistance and energy absorption performance in engineering practice.

Due to the excellent impact resistance of lightweight sandwich structures, numerous scholars have conducted in-depth research on their penetration performance. Park et al. [13] tested honeycomb-core sandwich plates, assessing their damage tolerance. Ying et al. [14] examined the influence of face sheet thickness, core height, and density on energy absorption performance. Amith et al. [15] conducted low-velocity impact tests to investigate the penetration resistance of foam-core sandwich composite structures, demonstrating that enhancing the anti-penetration capability of the composite structure can effectively mitigate damage caused by low-velocity impacts. Sun et al. [16] examined honeycomb sandwich plates with different structural parameters (face sheet thickness, core height, cell size, and wall thickness). Huo et al. [17] studied impactor shape and size effects on AFSPs, and identified penetration resistance mechanisms. Yang et al. [18] systematically investigated the influence of face-sheet thickness, core-layer thickness, and projectile geometry on the penetration resistance of sandwich plates, proposing a comprehensive classification system for typical penetration failure modes. Chen et al. [19] studied AFSPs under localized blast loading, and revealed effects of face sheet thickness and mass distribution on energy absorption. Sarkhosh et al. [20] integrated considerations of porosity and size effects in experimental and numerical studies on the ballistic performance of AFSPs, elucidating failure mechanisms and discussing the functional role of the foam core. Wang et al. [21] analyzed the dynamic response, energy absorption, and failure modes of sandwich plates during penetration based on drop weight impact test results.

In studying the penetration performance of sandwich structures, researchers have not only examined the influence of the parameters of sandwich plates but also further investigated temperature factors. Matthew et al. [22] investigated the effect of temperature (−25 °C, 25 °C, and 75 °C) on the impact behavior of composite sandwich plates, demonstrating that temperature significantly influences absorbed energy and maximum impact force. Gupta et al. [23] employed a shock tube apparatus to study the dynamic response of composite sandwich structures under varying temperatures, revealing that the structures exhibit brittle behavior when subjected to impact at subzero temperature conditions. Xi et al. [24] conducted low-velocity impact tests on AFSPs in high-temperature environments, and analyzed the evolution of failure modes, peak impact force, and energy absorption with temperature variations. Kaczyński et al. [25] performed impact experiments on AFSPs subjected to extreme temperatures and diverse loading conditions, finding that extreme subzero temperatures do not affect energy absorption. Additionally, Guo et al. [26] examined the dynamic behavior of AFSPs under repeated impacts at low temperature, and comparative analysis of penetration performance between the upper and lower face sheets was conducted.

Numerous studies have shown that AFSPs exhibit excellent performance in ship protection design. Numerous scholars have investigated methods to enhance the impact resistance of AFSPs, but studies on their penetration behavior remain limited. While the influence of impactor shape on impact response has been correspondingly explored, research on the effect of impactor size is notably lacking. Additionally, although many studies have examined the impact resistance of sandwich plates under high temperatures, there is a lack of research on the breakdown response of AFSPs at low temperature. In this study, impact experiments on AFSPs were conducted using the INSTRON 9350 Drop Tower, with a focus on investigating the deformation and failure modes of AFSPs under varying impact energies, and the dynamic response performance such as impact force and displacement, as well as the loading and unloading characteristics were analyzed. Furthermore, comparative analysis was performed to elucidate the penetration performance of AFSPs under different impactor diameters, as well as whether low temperature will have a certain impact on the mechanical properties of the material, which will affect the penetration performance of AFSPs.

## 2. Materials and Experiment Setup

**Figure 1:**
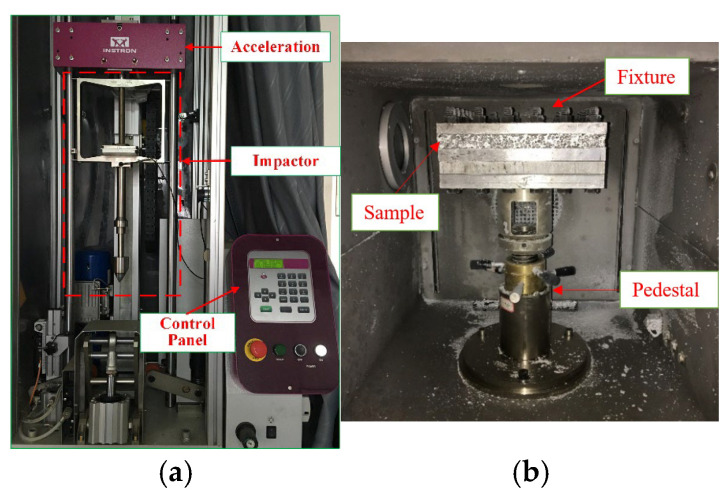
Experimental apparatus: (**a**) INSTRON 9350 Drop Tower; (**b**) environmental chamber.

The INSTRON 9350 Drop Tower was employed to conduct penetration experiments, as shown in Figure 1a. Instron 9350 impact testing system is primarily manufactured by Instron Corporation (also referred to as ITW Instron CEAST) in Pianezza, Italy.

The fixture consists of upper and lower clamps, which are bolted together with the AFSPs to simulate approximately fully clamped boundary conditions on all sides. As illustrated in Figure 1b, the drop hammer test device is equipped with an environmental chamber capable of controlling the ambient temperature of the sample, the temperature control range is −70 °C to 150 °C. Low temperatures are achieved using liquid nitrogen, while a temperature sensor inside the chamber monitors the real-time temperature to ensure stability during testing.

**Figure 2:**
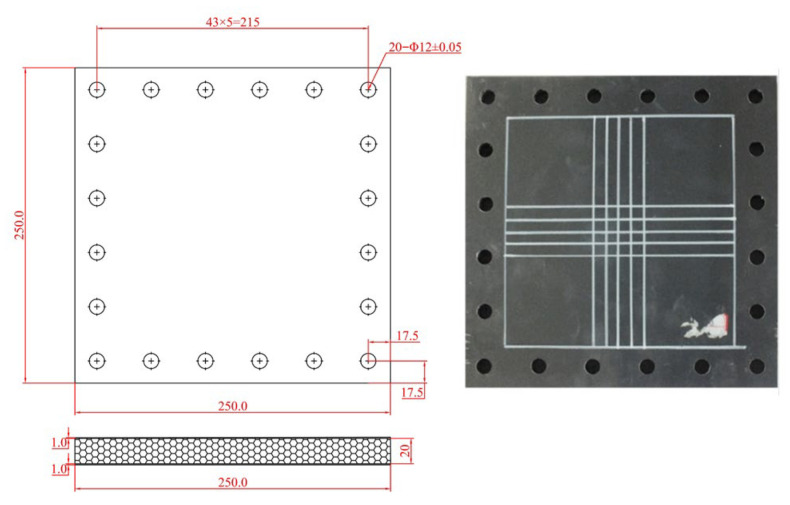
Sample of AFSPs.

The AFSPs sample, as shown in Figure 2, has overall dimensions of 250 mm × 250 mm with an effective internal area of 180 mm × 180 mm. The face sheet and the core layer are bonded with a self-prepared epoxy resin adhesive, and the epoxy resin and curing agent are modulated according to a 1:1 ratio to form an epoxy resin adhesive. Try to ensure that the amount of epoxy resin adhesive on the upper face sheet and lower face sheet is the same. The prepared epoxy resin adhesive is evenly applied to the face sheet, and then the core layer is placed between the upper and lower face sheet. Heavy objects were used to press on the upper face sheet to ensure close contact between the core layer and the face sheet. After the epoxy resin adhesive is cured, the manufacturing process of the aluminum foam sandwich plate is completed. A grid pattern with 10 mm spacing was marked at the center of each specimen to facilitate observation of deformation and failure modes. Each sample consists of three components: upper and lower face sheets with a thickness of 1 mm made of 5005H aluminum alloy, and a 20 mm thick aluminum foam core.

**Figure 3:**
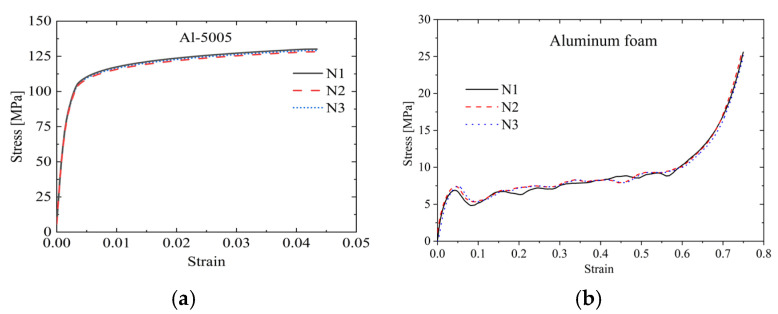
Stress–strain curve. (**a**) Tensile stress–strain curve of aluminum alloy; (**b**) compression stress–strain curve of aluminum foam.

To obtain the material parameters of the aluminum alloy (face sheet) and aluminum foam (core layer), compression and tensile tests were conducted using the Universal Testing Machine, respectively. Figure 3a presents the tensile stress–strain curve of the 5005H aluminum alloy, while Figure 3b shows the compressive stress–strain curve of the aluminum foam.

It can be seen from Figure 3. The material properties of the aluminum alloy and the aluminum foam are shown in Table 1. This study primarily investigates the effects of impact energy, impactor size, and a low-temperature environment on the penetration performance of AFSPs. The specific experimental conditions are detailed in Table 2.

## 3. Dynamic Behavior of AFSPs Under Low-Velocity Impact

### 3.1. Dynamic Responses

#### 3.1.1. Deformation and Failure Modes

As for the large impactor condition (D40), impact tests were conducted at four different energy levels: 200 J, 400 J, 500 J, and 600 J. The failure modes and dynamic responses of the AFSPs were analyzed under these varying impact energies.

**Figure 4:**
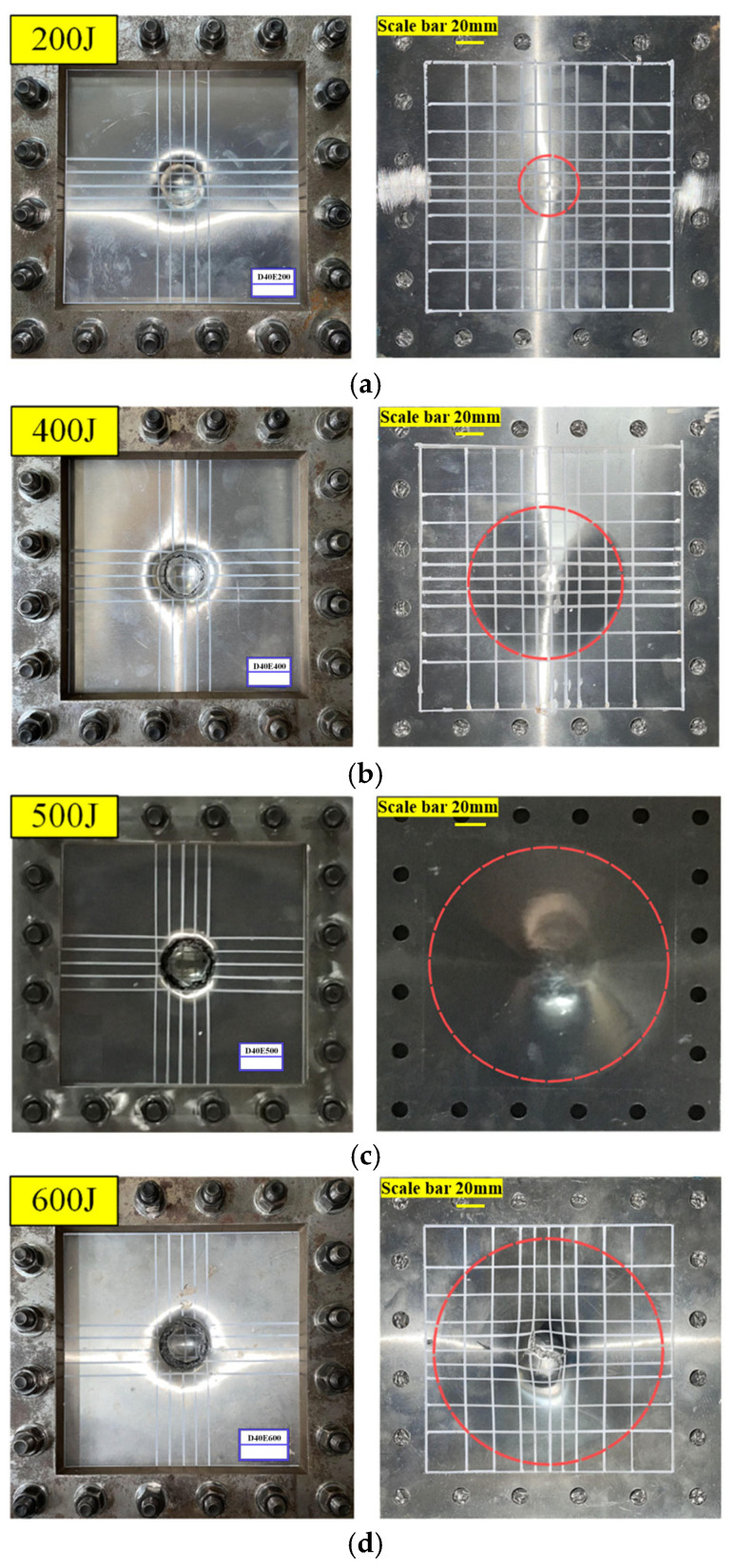
Deformation and failure of AFSPs under different impact energies at 20 °C. (**a**) Energy is 200 J; (**b**) energy is 400 J; (**c**) energy is 500 J; (**d**) energy is 600 J.

The deformation and failure of AFSPs under different impact energies at 20 °C is shown in Figure 4, and in this figure the dashed red circle represents the plastic hinge of the lower face sheet. At impact energy of 200 J, as shown in Figure 4a, the aluminum foam sandwich plate is subjected to local impact from the impactor, resulting in a high concentration of load in the impact area. For the upper face sheet, it is subjected to the downward concentrated force from the impactor and the upward distributed force from the aluminum foam core layer. Therefore, a shear phenomenon occurs in the circle around the contact area on the upper face sheet with the impactor, and the failure mode of the upper face sheet is a local shear failure, and it experiences local indentation with shear failure. The core layer underwent local compression, generating distributed loading on the lower face sheet, which consequently exhibited combined bending and tensile deformation with circular plastic hinge. When impact energy increased to 400 J, the upper face sheet showed local indentation with rupture, while the compressed core layer induced conical deformation in the lower face sheet under distributed loading. The plastic hinge progressively migrated toward boundaries with an expanding deformation zone as presented in Figure 4b. In addition, at an impact energy of 500 J, as shown in Figure 4c, the upper face sheet was completely broken off due to the shear effect, the core layer was compressed until was compacted, and the deformation area of the lower face sheet expanded to the boundary of the effective area of the AFSPs. Under 600 J impact, it can be seen from Figure 4d that the upper face sheet sheared off, the core compacted completely, and petal-shaped cracks developed in the lower face sheet from bending tensile failure. With the increase in impact energy, the failure mode of the upper face sheet transitioned from local indentation to fracture and detachment, with complete detachment occurring precisely at an impact energy of 500 J. The core layer gradually becomes compacted as the impact energy increases. The failure mode of the lower face sheet evolved from bending and tensile deformation to fracture with crack formation. At 500 J impact energy, the deformation zone of the lower face sheet extended to its boundary, while at 600 J, the lower face sheet experienced fracture with petal-shaped cracks.

From the above analysis, it can be observed that there is significant difference in the failure modes between the upper and lower face sheet of the AFSPs. The upper face sheet primarily undergoes shear failure, whereas the lower face sheet mainly experiences global bending and tensile fracture. As the impact energy increases, the deformation zone of the lower face sheet extends to the boundary of the effective region. Once the lower face sheet suffers tensile fracture, the AFSPs loses its loading capacity.

#### 3.1.2. Time History of Dynamic Responses

**Figure 5:**
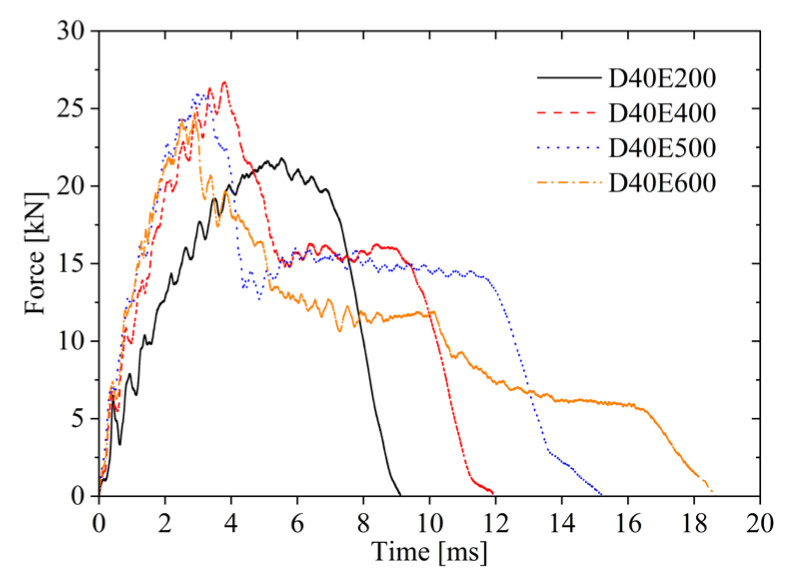
Force–time curves under different impact energies.

Figure 5 presents the impact force–time history curves under four different impact energy levels. At 200 J impact energy, the impact force increases progressively with time until reaching its peak value. Subsequently, as the elastic energy stored in the core layer is released, the impactor gradually separates from the upper face sheet, resulting in a decrease in impact force.

When the impact energy is 400 J, the impact force gradually increases over time until the upper face sheet fractures, reaching its peak value. The upper face sheet then loses its loading capacity, and the aluminum foam core layer undergoes continuous compression, forming a plateau region. Due to elastic energy release, the impactor rebounds and eventually detaches from the upper face sheet, causing the impact force to decrease until it reaches zero. It can be observed that without fracture of the lower face sheet, the impactor compresses the core layer, resulting in a plateau region in the impact force over a certain period. At 400 J, the core layer experiences partial compression, whereas at 500 J, the core layer becomes fully compacted, leading to a longer plateau region in the force–time curve. When the impact energy is 600 J, the upper face sheet fractures and detaches, and the aluminum foam core layer continues to compress, exhibiting an impact force plateau. Further compression of the core layer causes tensile fracture in the lower face sheet, leading to a sharp decline in impact force. As cracks propagate, the impact force decreases gradually until it diminishes to zero at approximately 18.5 ms.

**Figure 6:**
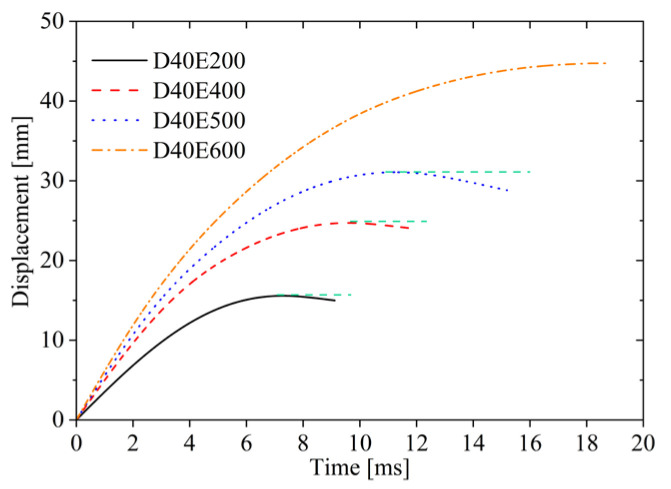
Displacement–time curves under different impact energy.

As shown in Figure 6, the displacement of the impactor increases over time, but the growth rate of the displacement curve gradually decreases until reaching the maximum displacement. In this figure, the dashed line represents the maximum value of the displacement. Higher impact energy results in larger displacement. As the impact energy increases, the growth rate of the displacement curve rises, and the time to reach maximum displacement extends, accompanied by the peak value of the displacement curve increasing. At impact energy of 200 J, the displacement grows with time, but due to the release of elastic energy stored in the AFSPs, partial deformation recovery occurs, leading to reduction in displacement; the final displacement is 0.8 mm less than the maximum displacement, namely a phenomenon known as “rebound”. Similarly, at 400 J, the release of stored elastic energy in the sandwich plates also causes rebound. Likewise, at 500 J, the displacement decreases after reaching its peak, and the extent of displacement reduction increases with higher impact energy; the final displacement of the D40E500 curve is reduced by 2.2 mm compared to the maximum displacement value, making the rebound effect more pronounced. However, at 600 J, due to the plastic failure caused by the fracture of the lower face sheet, there is no “rebound” phenomenon.

**Figure 7:**
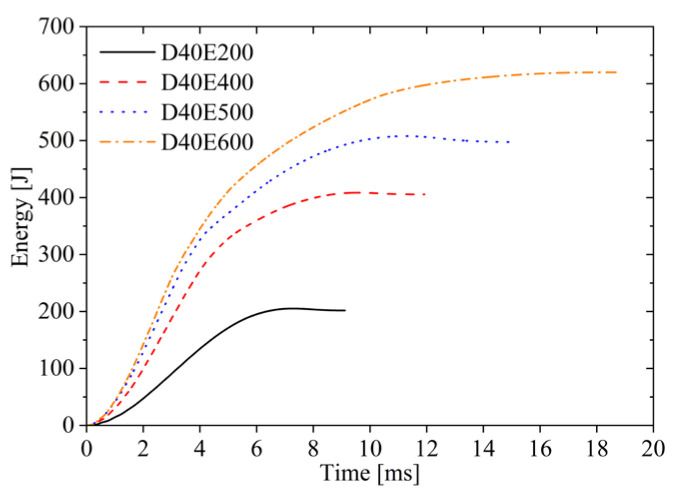
Energy–time curves under different impact energy.

During the impact process, the energy absorbed by the sandwich plate is defined as the absorption energy; the energy released by the elastic deformation of the sandwich plate is defined as the elastic energy; and the initial kinetic energy of the impactor is defined as the impact energy. The upper face sheet primarily absorbs energy through local indentation and fracture failure; the core layer absorbs energy through compression; and the lower face sheet absorbs energy through plastic deformation and fracture failure. As seen from Figure 7, the energy absorbed by the AFSPs increases over time, but the growth rate gradually decreases until reaching the maximum absorbed energy. At impact energies of 200 J, 400 J, and 500 J, the absorbed energy increases with time then decreases. Due to the release of elastic energy stored in the sandwich plates, the energy absorption continues decreasing until the impactor separates from the upper face sheet. However, when the impact energy is 600 J, due to the fracture of the lower face sheet, the final stage involves fracture energy absorption, and the energy absorption–time curve does not exhibit a descending phase.

#### 3.1.3. Performance of Loading and Unloading

**Figure 8:**
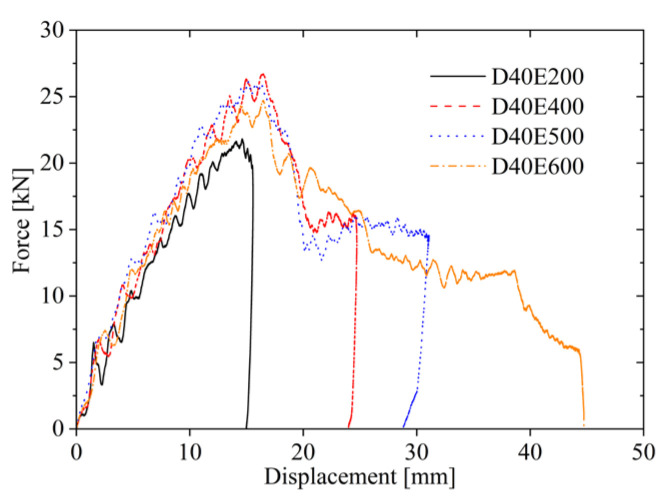
Force–displacement curve under different impact energy.

From Figure 8, it can be observed that at impact energy of 200 J, the impactor causes local indentation on the upper face sheet, resulting only in significant plastic deformation, and the force–displacement curve consists solely of loading and unloading phases. When the impact energy is 400 J, the upper face sheet is penetrated, and the impact force decreases due to friction as the impactor passes through it. As the core layer is compressed, the impact force stabilizes within a certain range, leading to a plateau in the force–displacement curve after the loading phase. Subsequently, the elastic energy stored in the AFSPs is released, causing the impactor to produce reverse displacement, reaching the unloading phase. When the impact energy is 500 J, no fracture occurs on the lower face sheet, and the plateau region extends further. At 600 J, both the upper and lower face sheets are penetrated. As the lower face sheet fractures, the plateau region in the force–displacement curve becomes longer compared to 500 J, and no unloading phase occurs.

**Figure 9:**
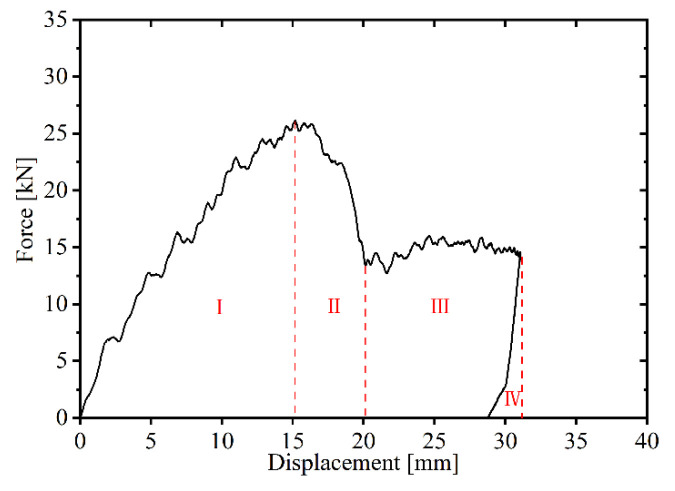
Four typical stages of force–displacement curve.

In the first stage (stage I), the force–displacement curves under all four energy levels are in the loading phase, exhibiting nearly identical loading stiffness. In the second stage (stage II), the impactor under the lowest energy separates from the upper face sheet during unloading, while those under the three higher energies penetrate the upper face sheet, with the impact force gradually decreasing due to friction. When the upper face sheet fractures while the lower face sheet remains intact, the force–displacement curve exhibits four typical stages, as shown in Figure 9. In the third stage (stage III), as the core layer compresses, the force–displacement curves for 400 J, 500 J, and 600 J display a plateau region. In the fourth stage (stage IV), the curves for 400 J and 500 J undergo unloading, whereas the 600 J impactor penetrates the lower face sheet. As illustrated in Figure 8, the 600 J force–displacement curve lacks an unloading phase, with the impact force gradually decreasing as displacement increases during lower face sheet penetration. The plateau region is shorter for 400 J but longer for 600 J, indicating that the plateau region expands with larger impact energy. The 400 J energy is insufficient for the impactor to contact the lower face sheet, whereas the 600 J impactor fully compacts the core layer and acts on the lower face sheet, causing its fracture and gradual decline in impact force with increasing displacement. In Figure 9, the red dashed line represents the value of displacement for the end of each stage.

### 3.2. Parameter Investigation on Penetration Responses

#### 3.2.1. Effect of Impactor Diameter

Under the impact energy of 400 J, the diameter of the impactor (including D25 and D40) was varied to comparatively analyze its effects on the failure mode, impact force, velocity, energy absorption, and loading and unloading performance of the AFSPs.

(1) Deformation and Failure Modes

**Figure 10:**
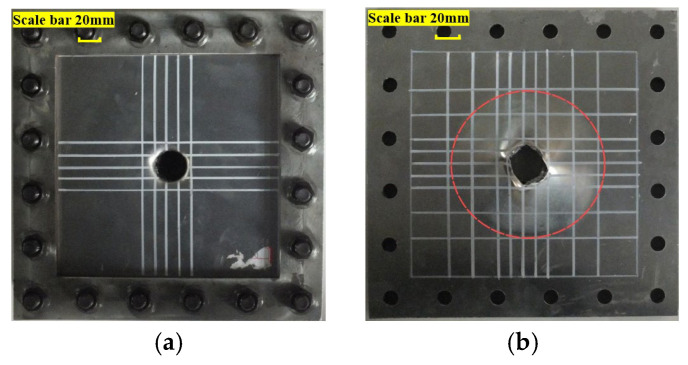

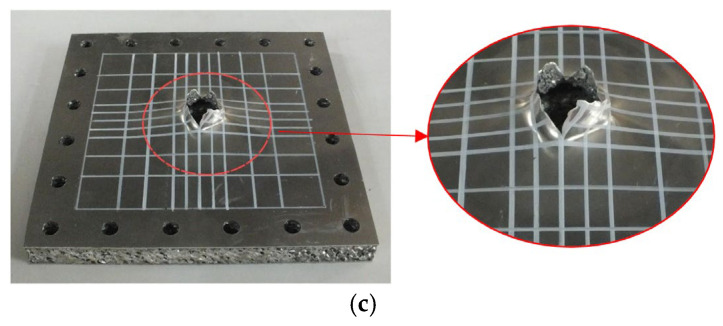
Failure of AFSPs under the action of D25. (**a**) Failure of front face sheet; (**b**) failure of lower face sheet; (**c**) rupture of lower face sheet.

At impact energy of 400 J with a 25 mm diameter impactor, the upper face sheet was completely penetrated by the impactor and fractured due to the shear, as shown in Figure 10a. The upper face sheet exhibited a relatively regular circular failure profile with minimal overall deformation. Figure 10b reveals that the lower face sheet underwent localized “petal-shaped” tearing failure with significant deformation concentrated within a circular area. The tearing area of the lower face sheet did not reach the boundaries. As seen in Figure 10c, the deformation of the lower face sheet was confined to the circular region, primarily due to the spherical shape of the impactor. In Figure 10, the dashed red circle represents the plastic hinge of the lower face sheet. Under loading stage, the lower face sheet experienced bending and tensile deformation, forming a circular plastic hinge. Both localized and overall deformations of the lower face sheet were evident, with evident tearing and detachment from the core layer. The aluminum foam core layer was partially penetrated but exhibited negligible overall deformation. This behavior is attributed to the concentrated force from the impactor acting on the upper face sheet, while the aluminum foam core exerted uniformly distributed pressure underneath, leading to shear failure in the upper face sheet. Due to the low shear failure strain, the fracture occurred easily, resulting in direct penetration with minimal global deformation.

(2) Time history performance

**Figure 11:**
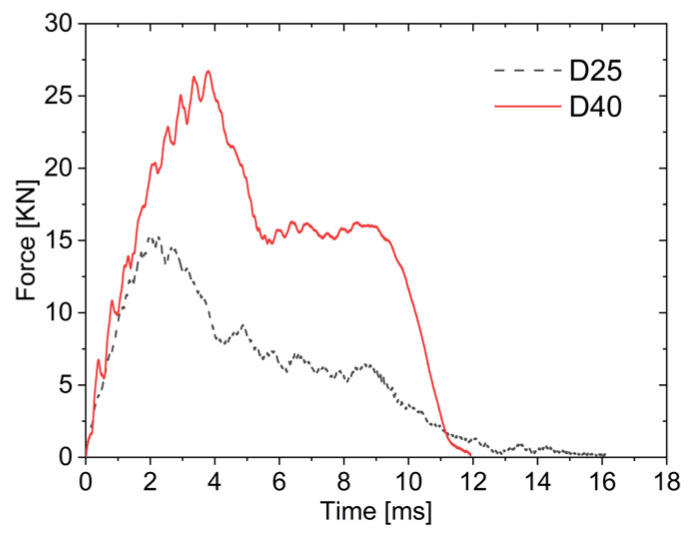
Force–time curves under different impactor diameters.

As shown in Figure 11, in the first stage, the impact forces of both the small (D25) and large (D40) impactors increase over time with nearly identical rates, but the peak impact force of the large impactor is significantly higher, which is almost double that of the small impactor. In the second stage, the impact forces decrease for both due to friction effects. During the third stage, as the core layer is compressed, the impact forces fluctuate within a narrow range, and both impactors exhibit plateau regions in their force–time curves. However, since the large impactor compresses a larger area of the AFSPs, its plateau effect is more pronounced. In the fourth stage, the impact force of the large impactor decreased rapidly as it separated from the upper face sheet. In contrast, the small impactor continued to act on the lower face sheet after core compaction until penetration occurred, resulting in a gradual decline in impact force–time history.

**Figure 12:**
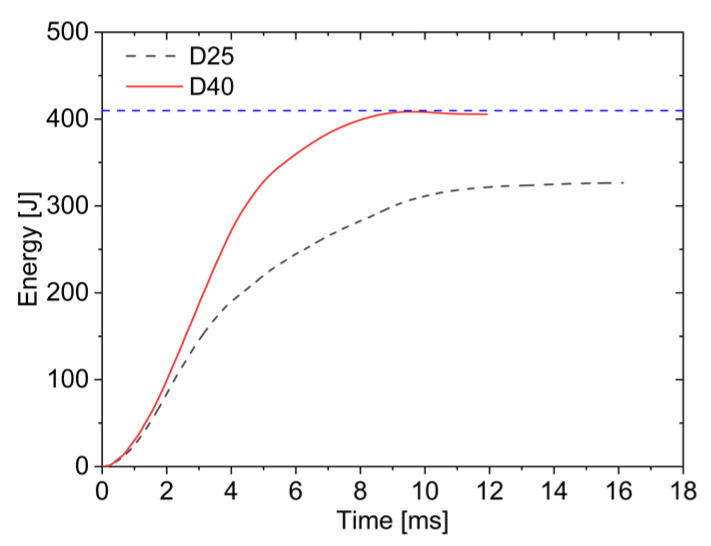
Energy–time curves under different impactor diameters.

The energy absorbed by the sandwich plates gradually increases with impact duration in Figure 12, with the energy absorption rate being higher for the D40 than the D25. In this figure, the blue dashed line represents the maximum value of the energy absorption. When using the D40, nearly all energy is absorbed by AFSPs. Since the lower face sheet remains unpenetrated under D40 impact, the stored elastic energy from core compression is released, causing a slight decrease in the energy absorption curve. In contrast, D25 retains residual velocity after penetrating the lower face sheet, with the AFSPs absorbing only 326.6 J of energy; the absorbed energy was reduced by about 18%. Smaller impactor diameters result in easier plate penetration and consequently lower energy absorption.

**Figure 13:**
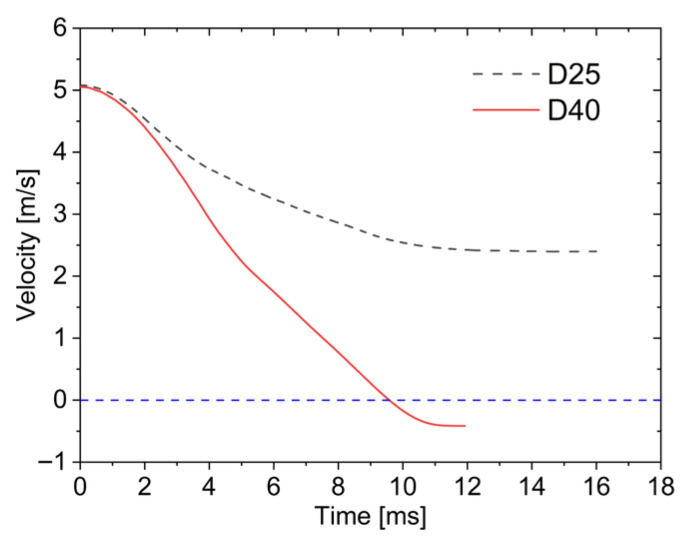
Velocity–time curves under different impactor diameters.

Figure 13 shows the time history curves of impact velocity for different impactor diameters. In this figure, the blue dashed line represent the special value of velocity, which is zero. It can be observed that larger impactors exhibit larger velocity reduction rates. For the D40, the impact velocity decreases rapidly over time. After velocity reaches zero, elastic energy stored in the sandwich plates is released, causing partial deformation recovery and the “rebound” phenomenon, where the impactor gains negative velocity (shown as negative values in the velocity curve). Under identical impact energy, the D25 penetrates the lower face sheet with gradually decreasing velocity, retaining a residual velocity of 2.4 m/s due to remaining energy after penetration.

(3) Loading–unloading performance

**Figure 14:**
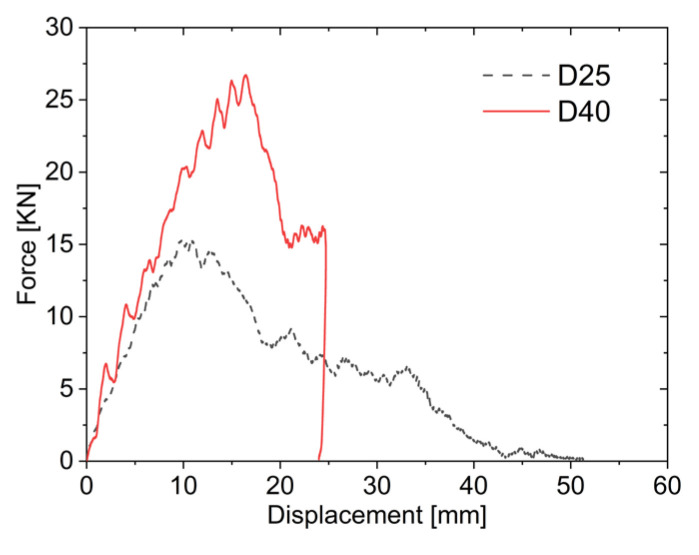
Force–displacement curves under different impactor diameters.

As illustrated in Figure 14, during the loading phase, the slope of the force–displacement curve for the larger impactor is slightly steeper, indicating that the loading stiffness of the larger impactor is greater than that of the smaller impactor. Subsequently, as the core layer is compressed, the force–displacement curve of the larger impactor exhibits a plateau phenomenon. Due to the larger diameter of D40, the contact area between the impactor and the sandwich plates is greater, resulting in a more pronounced plateau region. However, the residual impact energy after the fracture of the upper face sheet is relatively low, leading to a shorter plateau stage. In contrast, the contact area between D25 and the sandwich plates is smaller, and the reaction force from the aluminum foam core during compression is smaller. As a result, the impactor quickly comes into contact with the lower face sheet, which begins to provide resistance, leading to the plateau region showing less distinct. After the core layer is fully compacted, the impactor of D25 penetrates the lower face sheet. The impact of D25 on the lower face sheet causes its fracture, leading to gradual decline in impact force with increasing displacement, as energy is dissipated through friction between the impactor and the lower face sheet. Consequently, the impact force of the smaller impactor decreases progressively with displacement.

#### 3.2.2. Effect of Impact Energy

To analyze the effects of impact energy on the impact force, velocity, energy absorption, and loading–unloading performance of AFSPs, impact tests were conducted with a 25 mm diameter impactor at energy levels of 400 J and 800 J.

(1) Time history performance

**Figure 15:**
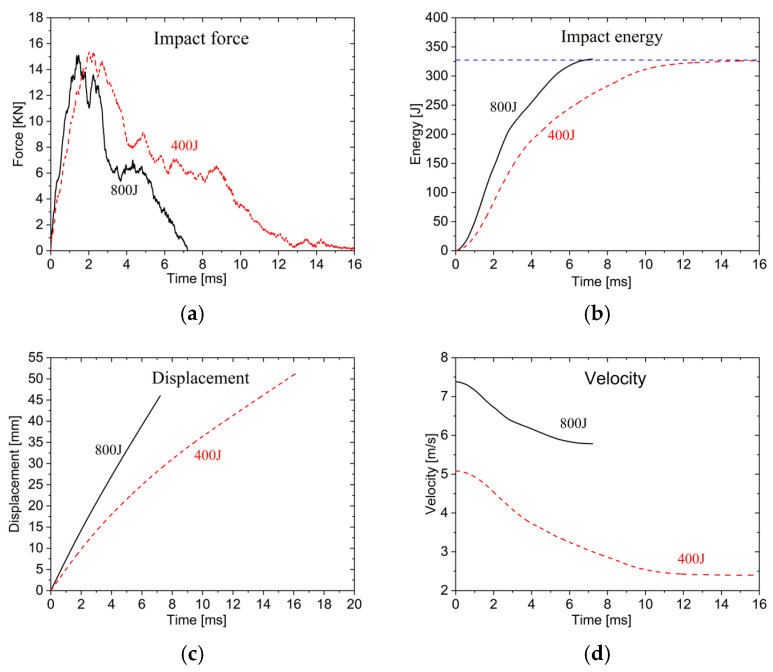
Time history curves under D25 impactor: (**a**) Impact force; (**b**) energy; (**c**) displacement; (**d**) impact velocity.

Figure 15a shows the impact force–time history curves for the two energy levels. In the first stage, as the impactor acts on the upper face sheet, the impact force increases rapidly. The 800 J curve exhibits a slightly larger growth rate than the 400 J curve, reaching its peak at 15.1 kN first, followed by the 400 J curve peaking at 15.3 kN. The impact energy mainly affects the time to reach peak force rather than the peak impact force. In the second stage, the impact force decreases due to the penetration of upper face sheet. The third stage involves gradual core layer compression. During the fourth stage, as the impactor acts on the lower face sheet, the impact force gradually decreases over time. Higher impact energy leads to faster force reduction to zero and earlier penetration.

Figure 15b presents the time history curve of energy absorption by the AFSPs. The absorbed energy increases gradually over time, with higher impact energy resulting in steeper energy absorption curves and faster absorption rates. However, the final energy absorption remains similar, indicating that under 800 J impact energy, significant residual energy remains unabsorbed. For small impactor impacts (i.e., D = 25 mm), the plates cannot fully absorb the impact energy, leading to complete penetration with residual velocity.

As seen from Figure 15c, displacement increases progressively with time, and higher impact energy produces faster displacement growth rates, though the penetration displacement remains essentially identical.

Figure 15d displays the impact velocity–time history curves. The impact velocity decreases gradually over time with diminishing reduction rates. Both energy levels result in residual velocities, with the 800 J impact exhibiting notably higher residual velocity.

(2) Loading–unloading performance

**Figure 16:**
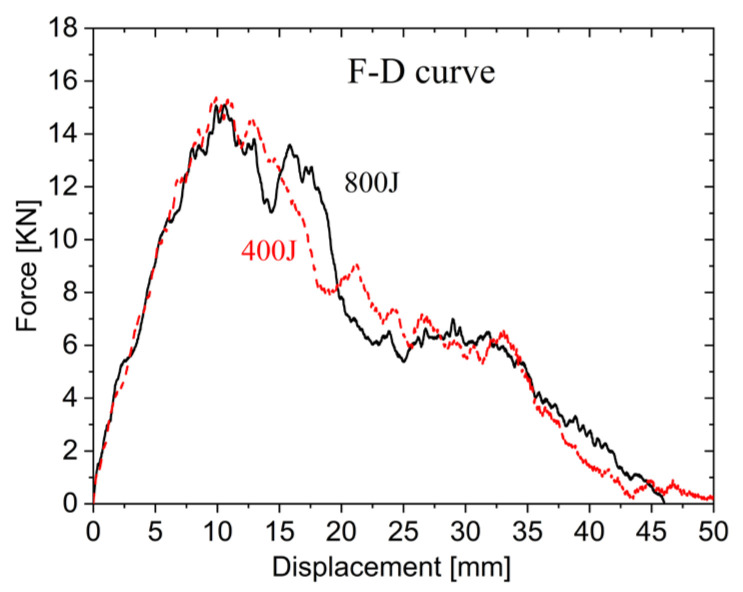
Force–displacement curves under D25 impactor.

Figure 16 compares the force–displacement curves at 400 J and 800 J impact energies. The loading phase demonstrates nearly identical stiffness for both energy levels, with similar displacements required to reach peak impact force. Core compression creates a plateau region, followed by gradual force reduction during lower face sheet penetration until complete dissipation. When using identical impactor diameters, the force–displacement performance (loading–unloading behavior) remains essentially unaffected by impact energy variations in cases of complete penetration.

#### 3.2.3. Effect of Ambient Temperature

To analyze the effect of low temperature on the penetration response of AFSPs, impact tests were conducted at 400 J impact energy with a 25 mm diameter impactor under different ambient temperatures, including 20 °C, −20 °C, −60 °C. This study investigated the influence of low temperatures on impact force, velocity, energy absorption, and loading–unloading performance.

(1) Time history performance

**Figure 17:**
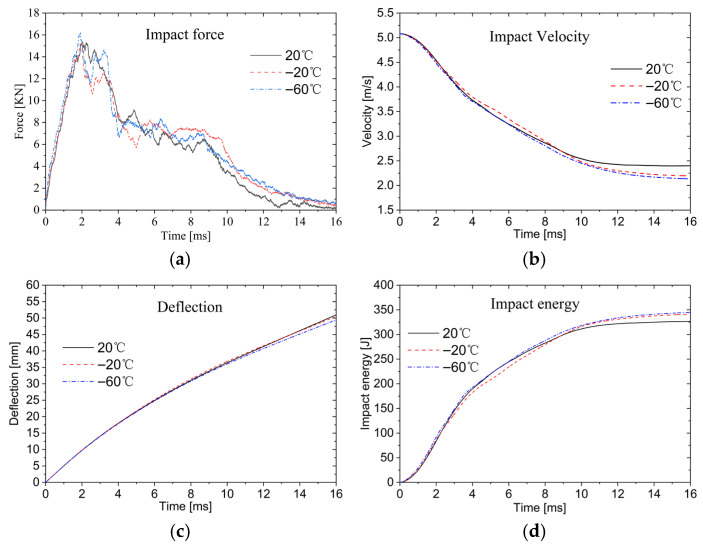
Time history curve of AFSPs at low temperature: (**a**) impact force; (**b**) impact velocity; (**c**) impact displacement; (**d**) energy.

As temperature decreases, both the aluminum alloy face sheets and aluminum foam core undergo transition from ductile to brittle. The yield strength of aluminum alloy at low temperature usually increases, but the fracture toughness decreases, and the fracture mode changes from ductile fracture to brittle fracture. The cell wall of aluminum foam will be brittle at a low temperature, which leads to the change in the overall compression performance of aluminum foam, that is, the platform stress may increase, but the compression failure gradually changes from plastic buckling to brittle fragmentation, and the energy absorption efficiency may be reduced. As shown in Figure 17a, under low-temperature conditions, the more brittle materials exhibit reduced deformability, resulting in higher impact forces required for upper face sheet fracture. The peak impact force is greatest at −60 °C. As temperature dropped to −60 °C, compared with the impact force peak at 20 °C, it increased by about 5.2%. The plateau regions during core compression remain nearly identical across different low-temperature conditions. When the lower face sheet fractures, the required impact force is smallest at 20 °C.

Figure 17b shows that, compared to the 20 °C impact velocity curve, the −20 °C and −60° curves eventually stabilize at lower values, indicating reduced residual velocities. Correspondingly, Figure 17d presents the energy absorption–time history curves. With decreasing temperature, the material undergoes transition from ductile to brittle behavior, making the sandwich plate more resistant to deformation. During the penetration process of the impactor, the energy absorbed by the sandwich plate increases, while the residual velocity of the impactor decreases accordingly. Compared with the normal temperature (20 °C), the energy absorption increases by about 8% at low temperature (−60 °C).

Figure 17c shows the deflection–time history curves under different temperature conditions. The results demonstrate that decreasing temperature causes a gradual transition from ductile to brittle behavior in the material, increasing its yield stress. Under identical impact energy, this leads to reduced permanent deflection of the sandwich plates.

(2) Load–Unload performance

**Figure 18:**
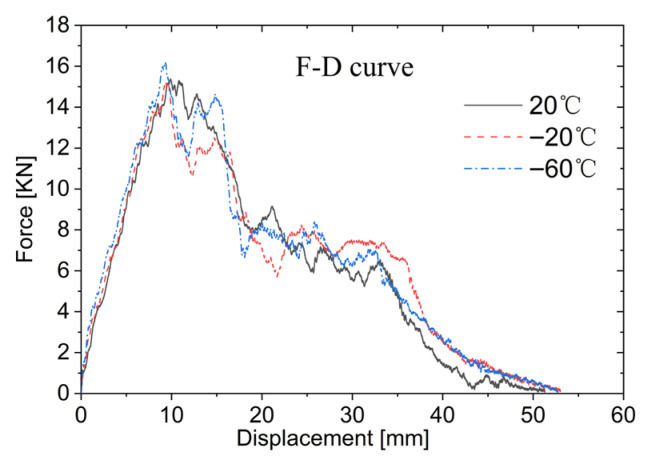
Force–displacement curves under low temperature.

**Figure 19:**
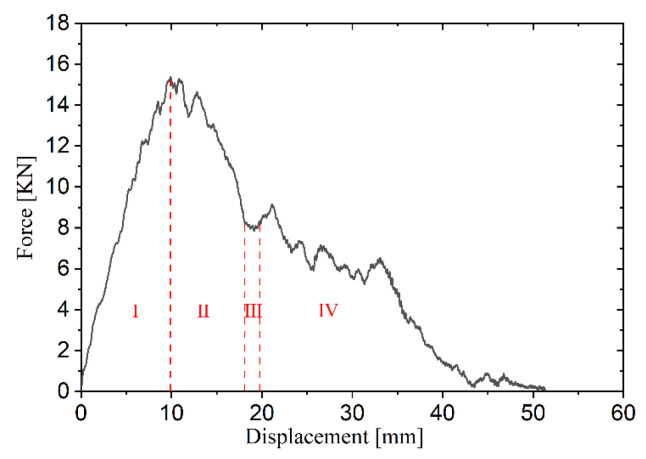
Four typical stages of force–displacement curve for penetration cases.

Figure 18 displayed the force–displacement curves at 20 °C, −20 °C, and −60 °C, respectively. For penetration cases, the loading–unloading behavior can be divided into four typical stages as illustrated in Figure 19. The force–displacement curves under different temperature all exhibit a loading phase in the first stage (stage I), where the loading stiffness increases slightly as the temperature decreases. The peak impact forces at 20 °C and −20 °C are relatively similar, whereas at −60 °C, the peak impact force shows a noticeable increase; compared to 20 °C, it increases by about 5.2%. In the second stage (stage II), the impact force decreases as the displacement continues to rise. The third stage (stage III) reveals a plateau region corresponding to the core compression, and the influence of low temperatures on this plateau phenomenon is not significant. In the fourth stage (stage IV), the impactor penetrates the lower face sheet, leading to reduction in impact force with further displacement.

## 4. Conclusions

In this paper, the impact tests on AFSPs were conducted using INSTRON 9350 Drop Tower, and the deformation mode, failure mode, impact force, load–unload performance of AFSPs were analyzed. Meanwhile, the influences of impactor diameter, impact energy, and ambient temperature on penetration performance were explored. The main conclusions are as follows:

(1) With different impact energies, the deformation and failure process of face sheet and core layers was first involved in local indentation and shear failure of the upper face sheet, followed by gradual compression of the core layer, which finally turned into a bending tensile deformation developing into fracture failure of the lower face sheet. Before the lower face sheet experienced fracture, displacement increased with impact energy, and the “rebound” phenomenon became more obvious. Once the lower face sheet experienced fracture, deformation could not recover, and no “rebound” phenomenon occurred. Regarding loading–unloading performance under different impact energies, in the first stage, the force–displacement curves were all in the loading stage with almost identical loading stiffness. In the second stage, the 200 J impactor separated from the upper face sheet during unloading, while the other three energy levels showed plateau phenomena in their force–displacement curves. In the third stage, only the 600 J impact energy curve did not undergo an unloading stage due to the impactor penetrating the lower face sheet.
(2) The diameter of the impactor has a certain influence on the dynamic responses of AFSPs. Under an impact energy of 400 J, the D40 exhibits higher peak impact force, almost twice as much as the D25. In terms of the force–displacement curve, the D40 separates from the sandwich plates and undergoes an unloading phase, whereas the D25 penetrates the lower face sheet without an unloading stage. As for the D40, nearly all the energy was absorbed by the sandwich plates, whereas the smaller impactor diameter led to easier penetration of the sandwich plates and only 326.6 J was absorbed by D25; the absorbed energy was reduced by about 18%. After penetrating the sandwich plates, the D25 retains residual velocity. However, for the D40, once the velocity decreases to zero, the “rebound” phenomenon occurs due to partial elastic recovery of the deformed structure, resulting in a reverse velocity value.
(3) During impact, the upper face sheet mainly absorbed energy through local indentation and fracture failure, the core layer mainly absorbed energy through compression, and the lower face sheet mainly absorbed energy through bending tensile deformation and fracture failure. Due to the release of elastic energy stored in the sandwich plates, the “rebound” phenomenon occurred. The higher the impact energy, the more obvious the plateau phenomenon in the force–displacement curve. Secondly, in the case of D25, different impact energies affected the time taken to reach the peak impact force but had insignificant effects on peak impact force magnitude. Higher impact energies resulted in faster energy absorption rates, but the final absorbed energy values were almost identical. Under D25 conditions, different impact energies had minor effects on loading–unloading performance.
(4) At 400 J impact energy with D25, as temperature decreased, both the aluminum alloy face sheet and aluminum foam core layers transitioned from ductile to brittle behavior, and yield stress increased, leading to reduced permanent deflection of the face sheets. As temperature dropped to −60 °C, compared with the impact force peak at 20 °C, it increased by about 5.2%, and the sandwich plates absorbed more energy, while residual velocity decreased. The first stage under different temperatures was always the loading stage, with loading stiffness slightly increasing as temperature decreased, while low temperature had insignificant effects on plateau phenomena. In the future, scanning electron microscopy and industrial CT scanning can be further applied to obtain the failure image inside the aluminum foam sandwich plate, so as to more intuitively analyze the failure mechanism of the aluminum foam sandwich plate under penetration state.

## Figures and Tables

**Table 1:** Material properties.

Material	Density (g/cm^3^)	Elastic Modulus (GPa)	Yield Strength (MPa)	Plateau Stress (MPa)	Densification Strain
Al-5005	2.7	34	102	-	-
Aluminum foam	0.44	0.161	-	7.4	0.65

**Table 2:** The impact cases of AFSPs.

Impact Case	Impactor Diameter (mm)	Impact Energy (J)	Ambient Temperature (°C)	Impact Mass (kg)	Impact Velocity (m/s)
D25E400	25	400	20	31.029	5.08
D25E800	25	800	20	31.029	7.18
D25E400T20	25	400	20	31.029	5.08
D25E400T-20	25	400	−20	31.029	5.08
D25E400T-60	25	400	−60	31.029	5.08
D40E200	40	200	20	31.439	3.57
D40E400	40	400	20	31.439	5.05
D40E500	40	500	20	31.439	5.64
D40E600	40	600	20	31.439	6.18

(Note: In this paper, D25 represents a 25 mm diameter impactor, E400 denotes 400 J impact energy, and T20 indicates a test temperature of 20 °C for simplified description).

## Data Availability

The original contributions presented in this study are included in the article. Further inquiries can be directed to the corresponding author.

## Abbreviations

The following abbreviations are used in this manuscript:

AFSPs	Aluminum foam sandwich plates
D25	25 mm diameter impactor
D40	40 mm diameter impactor
